# Association of Hemoglobin to Red Blood Cell Distribution Width Ratio and Total Bone Mineral Density in U.S. Adolescents: The NHANES 2011–2018

**DOI:** 10.3390/diagnostics15202567

**Published:** 2025-10-12

**Authors:** Tianhao Guo, Jiheng Xiao, Xinjun Yao, Jiangbo Bai, Yadong Yu

**Affiliations:** 1 Department of Hand Surgery, Hebei Medical University Third Hospital, Shijiazhuang 050051, China; 25032100244@stu.hebmu.edu.cn (T.G.);; 2 Department of Orthopaedics, Union Hospital, Tongji Medical College, Huazhong University of Science and Technology, Wuhan 430022, China

**Keywords:** adolescents, skeletal development, hematological biomarkers, pediatric population, bone health, osteoporosis risk

## Abstract

**Background**: The hemoglobin-to-red-cell distribution width ratio has emerged as a novel prognostic marker in various clinical settings. However, its association with total bone mineral density in adolescents remains inadequately explored. **Methods**: This cross-sectional study was based on data from the 2011–2018 National Health and Nutrition Examination Survey, including adolescents aged 12–19 years with complete data on hemoglobin, red cell distribution width, and total bone mineral density. Weighted multivariable linear regression models and generalized additive models were used to evaluate the association between hemoglobin-to-red-cell distribution width and total bone mineral density. A two-piecewise linear regression model was applied to assess potential threshold effects, with log-likelihood ratio tests used to determine the significance of inflection points. Subgroup and interaction analyses were further conducted to examine whether age, sex, race, and milk product consumption modified this association. **Results**: A total of 3789 adolescents were included. Participants in the highest hemoglobin-to-red-blood-cell distribution width ratio quartile had significantly higher hemoglobin levels, lower red blood cell distribution width, greater total bone mineral density, higher total calcium and blood urea nitrogen levels, and lower body mass index, high-density lipoprotein cholesterol, and serum 25OHD levels compared to lower quartiles. The hemoglobin-to-red-blood-cell distribution width ratio was positively associated with total bone mineral density (fully adjusted β = 0.078, 95% CI: 0.053, 0.104, *p* < 0.0001). A two-piecewise linear regression model identified an inflection point at the hemoglobin-to-red-cell distribution width ratio = 1.055; the positive association became stronger above this threshold (β = 0.143 vs. β = 0.039 below the threshold, *p* = 0.003 for nonlinearity). Subgroup analysis revealed significant gender interactions (*p* < 0.0001). A higher HRR was significantly associated with greater total BMD in males (β = 0.130, 95% CI: 0.089–0.171, *p* < 0.0001), whereas no significant association was observed in females (β = −0.009, 95% CI: −0.043–0.025, *p* = 0.604). Positive associations were also observed among participants aged 12–15 years, non-Hispanic Whites, non-Hispanic Blacks, other Hispanics, Mexican Americans, and frequent milk consumers. **Conclusions**: Our results indicate that the hemoglobin-to-red-cell distribution width ratio shows a potential association with bone mineral density in male adolescents, which may offer supportive value for bone health assessment but requires further validation.

## 1. Introduction

Bone mineral density (BMD) is a crucial indicator of skeletal health, reflecting bone strength and quality. In adolescents, achieving a high BMD is especially important because nearly 90% of peak bone mass is accrued by around 18 years of age [1,2]. Optimizing bone health during this window can yield lifelong benefits. This peak bone mass often determines the risk of osteoporosis and fragility fractures later in life [3]. Thus, bone density in youth has significant long-term health implications, and understanding factors that influence BMD during adolescence is a public health priority.

Hemoglobin (HGB) and red cell distribution width (RDW) are two key hematological indices that provide insight into systemic health beyond their basic physiological roles. HGB is the oxygen-carrying protein in red blood cells [4], and low HGB (anemia) is common in various pathological conditions (e.g., chronic inflammatory diseases and nutritional deficiencies) [5,6,7,8,9]. Anemia has been recognized by most researchers as a risk factor for impaired bone health: people with low HGB tend to experience lower BMD, higher fracture incidence, and greater risk of osteoporosis [10]. For example, a large cohort study in adults over 40 found that individuals with lower HGB levels had significantly higher odds of developing osteoporosis, even after adjusting for confounders [11]. Another study pointed out that BMD in adolescents with thalassemia was significantly lower than in healthy individuals of the same age [12]. Collectively, these findings suggest that pathological reductions in HGB—particularly those associated with chronic disease or nutritional deficiency—may have detrimental effects on bone density and osteoporosis risk. While most studies support this negative association, some have reported non-significant findings [13], indicating possible variability across populations or study designs. RDW is routinely assessed with a complete blood count [14]. RDW is a measure of the variation in red blood cell size and is often elevated in some disease states [14,15,16]. In contrast to HGB, RDW increases in response to disrupted erythropoiesis caused by nutritional deficiencies, systemic inflammation, or chronic illness [17,18]. Elevated RDW has been observed in individuals with liver disease, autoimmune disorders, malignancies, and during post-transfusion recovery [16,19,20,21]. Several studies have demonstrated associations between high RDW and adverse skeletal outcomes. A study of elderly men reported that men in the highest quartile of RDW had about a 2.8-fold greater risk of hip fractures compared to those in the lowest quartile [22]. Another study showed that there is a negative correlation between RDW and femoral neck BMD, and this correlation exists in both men and postmenopausal women [23]. These findings suggest that elevated RDW may reflect inflammatory or metabolic imbalances that negatively influence bone metabolism. Although RDW has shown clinical relevance in adult health assessments, its relationship with BMD in adolescents remains insufficiently investigated.

The hemoglobin-to-red-blood-cell distribution width ratio (HRR) has emerged as a novel inflammatory marker. The HRR, easily calculated from routine blood counts, is recognized as an inflammatory index associated with the incidence and adverse outcomes of various diseases [24,25,26]. The HRR is easily calculated from routine blood tests and is thought to integrate oxygen transport capacity and red cell variability, offering a more stable and comprehensive indicator of systemic health than either component alone [27,28,29]. The HRR has been associated with adverse outcomes in a range of diseases. For instance, a diminished HRR has shown prognostic value for poorer survival in cancers such as esophageal squamous cell carcinoma and worse long-term outcomes in cardiovascular conditions [30,31]. In patients with heart failure and stroke, a lower HRR correlates with higher rates of readmission or unfavorable prognosis, underscoring its role as a meaningful inflammatory index [32,33,34]. Despite its growing recognition in oncology and cardiovascular research, the role of the HRR in bone metabolism is only beginning to be explored. A recent study demonstrated that individuals with a higher HRR had significantly greater femoral BMD, suggesting that the HRR may serve as a useful integrative marker of skeletal health and fracture risk in older adults. Given that chronic inflammation can impair bone remodeling by promoting osteoclast activity and suppressing osteoblast function, the HRR may reflect systemic inflammatory conditions that influence bone mineralization [35,36]. However, no studies to date have systematically examined the association between the HRR and BMD in adolescents. Therefore, the present study aims to investigate the relationship between the HRR and BMD in U.S. adolescents, with the goal of providing theoretical evidence and practical insights for early intervention strategies targeting bone health during this critical developmental period.

## 2. Methods

### 2.1. Data Source and Study Population

**Figure 1:**
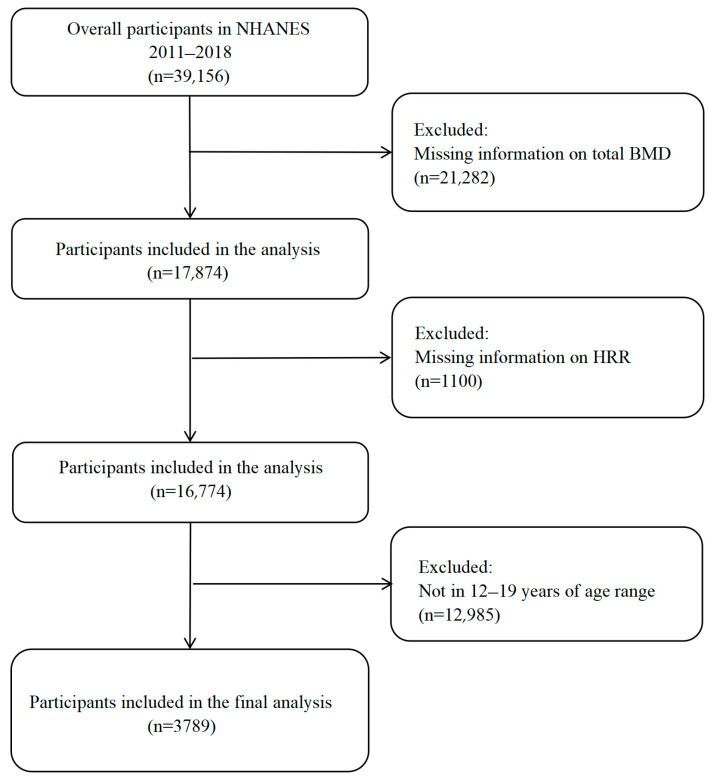
Flow chart of participants selection.

The National Health and Nutrition Examination Survey (NHANES) is an ongoing, large-scale cross-sectional survey conducted in the United States to provide objective health data and address emerging public health challenges in the general population. The survey methods were approved by the Institutional Review Board of the National Center for Health Statistics, and all participants in NHANES provided consent for their data to be used in the study. The present analysis was performed in accordance with the ethical standards laid down in the 1964 Declaration of Helsinki and its later amendments. This study utilized NHANES datasets collected between 2011 and 2018. A total of 39,156 participants were enrolled in NHANES from 2011 to 2018. For this analysis, we first excluded participants without valid total BMD data (*n* = 21,282) and those missing HRR values (*n* = 1100). We further restricted the age range to 12–19 years, excluding 12,985 participants outside this range. The final analytic sample consisted of 3789 adolescents (Figure 1).

### 2.2. Study Variables

HRR was calculated as the ratio of HGB(g/dL) to RDW (%) [37]. As a ratio of these two hematological parameters, the HRR is unitless. Blood specimens were measured at the NHANES Mobile Examination Centers (MECs). Detailed specimen collection and processing instructions are discussed in the NHANES Laboratory/Medical Technologists Procedures Manual (LPM). The Beckman Coulter MAXM instrument in the MECs produces a complete blood count on blood specimens and provides a distribution of blood cells for all participants. A detailed description of the laboratory method used can be found on the NHANES website.

According to the NHANES protocol, participants aged 8–59 years were eligible for whole-body dual-energy X-ray absorptiometry (DXA) scans. Pregnant females were excluded from the DXA examination. Additional exclusion criteria included recent use of radiographic contrast material (barium) within 7 days, self-reported weight over 450 pounds, or height greater than 6′5″, due to technical limitations of the DXA equipment.

### 2.3. Covariates

To minimize the influence of confounding factors on the relationship between the HRR and total BMD, multiple covariates were included in the adjusted models. The selection of these covariates was guided by previous NHANES-based studies and supported by expert consensus in the literature. The final model included age, gender, race, ratio of family income to poverty (PIR), total calcium, high-density lipoprotein cholesterol (HDL-C), serum 25-Hydroxyvitamin D (serum 25OHD), milk product consumption, body mass Index (BMI), and blood urea nitrogen (BUN) as covariates. Serum 25-Hydroxyvitamin D is the sum of 25-Hydroxyvitamin D2 and 25-Hydroxyvitamin D3. Detailed information on variable definitions and data collection procedures can be found in the NHANES Methods and Analysis Guide (https://wwwn.cdc.gov/nchs/nhanes/AnalyticGuidelines.aspx, accessed on 10 March 2025).

### 2.4. Statistical Methods

In descriptive analyses, continuous variables were expressed as means ± standard deviations and compared using weighted linear regression, while categorical variables were presented as percentages and compared using the chi-square test. To investigate the associations of RDW and HRR with total BMD, multivariable regression models accounting for the complex sampling design of NHANES (sampling weights) were employed. Three models were constructed: Model 1 was unadjusted; Model 2 was adjusted for gender, age, and race; and Model 3 was further adjusted for age, gender, race, PIR, total calcium, HDL-C, serum 25OHD, milk product consumption, BMI, and BUN. In the sensitivity analysis, HRR was converted from a continuous variable to a categorical variable (quartiles) to assess the robustness of the findings. In addition, a generalized additive model (GAM) and smooth curve fitting were utilized to further explore the relationship between HRR and BMD. A two-piecewise linear regression model (segmented regression model) was applied to fit different intervals and estimate the threshold effect. A log-likelihood ratio test was used to compare the one-line (non-segmented) model with the two-piecewise linear model in order to determine whether a statistically significant threshold (inflection point, K) existed. To explore potential effect modification, subgroup analyses were conducted according to age, gender, race, and frequency of milk product consumption. Interaction terms were included in the models, and *p*-values for interaction were calculated to evaluate the heterogeneity of associations across subgroups.

We utilized the EmpowerStats (version 4.2) (www.empowerstats.com, accessed on 10 March 2025), which integrates multiple R packages to perform descriptive statistics, regression modeling, and smooth curve fitting.

## 3. Results

### 3.1. Study Population Characteristics

As depicted in Table 1, a total of 3789 participants were included in this study. The mean age was 15.406 ± 2.237 years. There were 2005 males and 1784 females. Participants were divided into quartiles based on HRR values (Q1–Q4) to compare characteristics (Q1: <0.9716; Q2: 0.9716–1.0635; Q3: 1.0635–1.1515; Q4: >1.1515). Between the HRR quartiles, there were significant differences in baseline characteristics. Individuals in the highest HRR quartile were more likely to be male and non-Hispanic White, with higher HGB, lower RDW, and greater BMD compared to other quartiles. Additionally, they exhibited higher serum total calcium, and BUN levels, whereas BMI, HDL-C, and serum 25OHD levels were lower in this group. Moreover, participants in the highest HRR quartile were more likely to consume milk products more frequently than those in the lower quartiles. To better quantify baseline differences across HRR quartiles, we reported standardized mean differences (SMDs) in addition to *p*-values in Table 1. 

### 3.2. Association Between RDW, HRR and BMD

**Figure 2:**
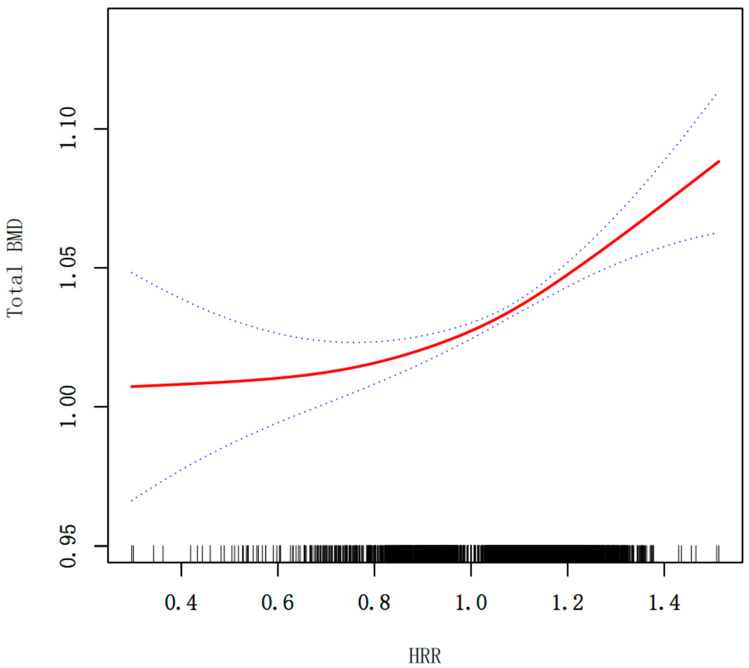
Smooth curve fitting for HRR and total BMD (g/cm^2^). The relationship between HRR and BMD was tested by the generalized additive model. Blue bands represent the 95% confidence interval from the fit.

Multivariate regression analyses (Table 2) revealed a significant positive association between HRR and BMD across all models. In the unadjusted model (Model 1), HRR was positively correlated with BMD (β = 0.105, 95% CI: 0.079, 0.131; *p* < 0.0001). This association remained robust after adjusting for age, gender, and race in Model 2 (β = 0.068, 95% CI: 0.043, 0.093; *p* < 0.0001). In the fully adjusted model (Model 3), which additionally adjusted for BMI, total calcium, HDL-C, serum 25OHD, milk product consumption and BUN, the positive association between HRR and BMD was slightly attenuated but remained statistically significant (β = 0.078, 95% CI: 0.053, 0.104; *p* < 0.0001). Moreover, when HRR was divided into quartiles, participants in the highest quartile (Q4) exhibited significantly greater total BMD compared to those in the lowest quartile (Q1), even after full adjustment. A significant linear trend was also observed across quartiles (*p* for trend <0.001), supporting a dose–response relationship between HRR and BMD (Table 2). Smooth curve fittings of the association between HRR and total BMD were shown in Figure 2. To further evaluate the potential association between HRR and total BMD, we performed an analysis using a two-piecewise linear regression model. An inflection point was identified at HRR = 1.055. When HRR was below the point, the association with total BMD was relatively modest (β = 0.039, 95% CI: 0.002, 0.076, *p* = 0.0405). In contrast, when HRR exceeded the point, the association became more pronounced (β = 0.143, 95% CI: 0.094, 0.193, *p* < 0.0001). The log-likelihood ratio test (*p* = 0.003) indicated a significantly better fit for the piecewise model compared to the standard linear model, supporting a nonlinear relationship (Table 3).

### 3.3. Subgroup Analysis

**Figure 3:**
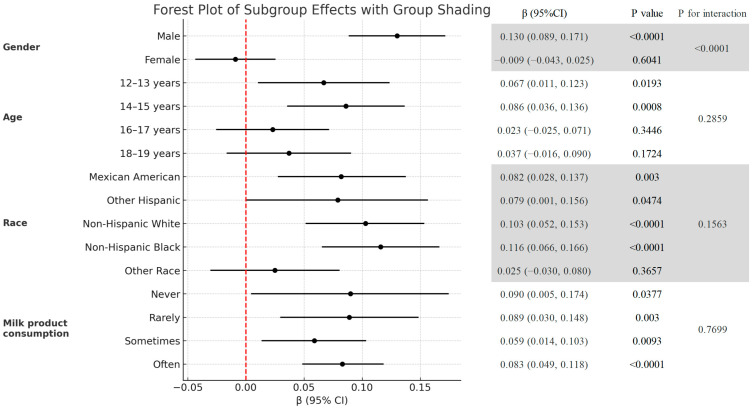
Subgroup analysis of the association between the HRR and total BMD (g/cm^2^).

**Figure 4:**
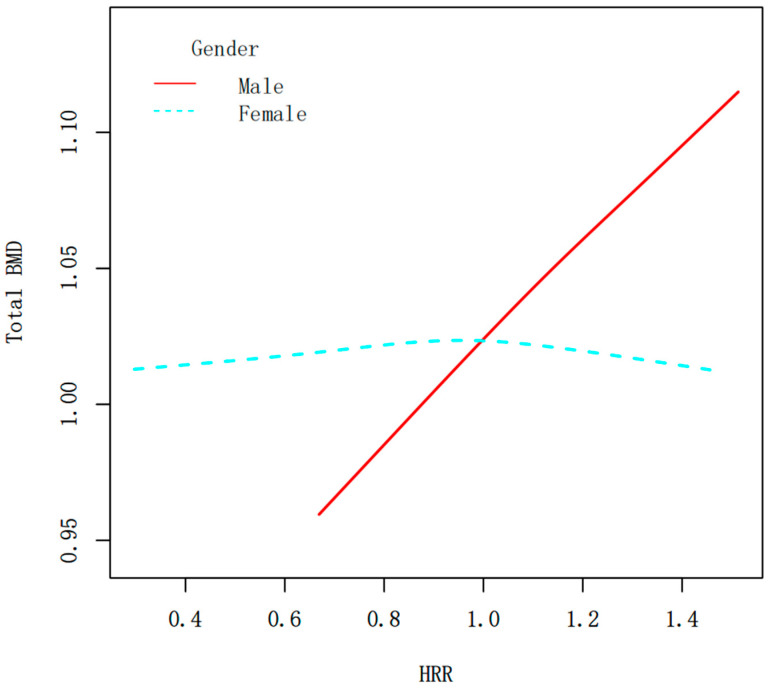
Smooth curve fitting of the HRR and total BMD (g/cm^2^) stratified by sex. The relationship between HRR and BMD was tested by the generalized additive model.

Figure 3 is the association between the HRR and total BMD stratified by age, gender, race, and milk consumption frequency. Stratified analyses revealed a significant interaction by gender (*p* for interaction < 0.0001) (Figure 3). In males, HRR exhibited a strong positive correlation with BMD, indicating that higher HRR levels were consistently associated with increased total BMD (β = 0.130, 95% CI: 0.089, 0.171, *p* < 0.0001). In females, HRR had no significant relationship with BMD (β = −0.009, 95% CI: −0.043, 0.025, *p* = 0.6041). Figure 4 shows the smoothed curve fit of the association between the HRR and total BMD stratified by sex (Figure 4). In addition, significant positive associations between HRR and total BMD were detected in subjects aged 12–15 years, non-Hispanic whites, non-Hispanic blacks, other Hispanics, Mexican Americans, and all milk drinkers (Figure 3).

## 4. Discussion

To our knowledge, this study is the first to investigate the association between the HRR and total BMD among adolescents using nationally representative data from NHANES 2011–2018. The results of this study indicate that, after adjusting for relevant confounding factors, a higher HRR was significantly associated with greater total BMD in male adolescents, while no significant association was observed in females.

Several biological mechanisms could explain the observed positive correlation between HRR and total BMD. First, HGB is crucial for oxygen transport; higher HGB levels improve tissue oxygenation, including in bone, and have been associated with better bone quality in prior studies [38]. Adolescents with higher HGB may also have greater exercise capacity, leading to more physical activity and mechanical loading on bone. Regular weight-bearing exercise is known to stimulate bone accretion [39]. Thus, a higher HRR (reflecting higher HGB) might partially indicate a lifestyle with more physical activity, which in turn promotes higher BMD. Second, a higher HRR also implies a lower RDW. RDW increases in the presence of chronic inflammation, oxidative stress, and malnutrition, factors that are detrimental to bone health [40,41,42]. Chronic systemic inflammation, for example, stimulates bone resorption and inhibits bone formation, contributing to loss of bone mass [43]. Consistently, inflammation and oxidative stress are recognized drivers of osteoporosis and low BMD [44,45]. Malnutrition (including deficiencies in calcium or vitamin D) likewise impairs bone development. Indeed, adequate nutritional status is essential for maintaining BMD [45]. A higher HRR may serve as a composite marker of better nutritional and inflammatory status, whereas a low HRR might signal underlying anemia or chronic inflammation that could compromise bone accrual. In summary, HRR encapsulates key hematologic and systemic factors—oxygen-carrying capacity, inflammatory burden, and nutritional state—all of which play important roles in bone metabolism. Thus, a higher HRR likely fosters a more favorable environment for bone growth and maintenance, whereas a lower HRR may reflect conditions that predispose to lower BMD and frailer bones. This interpretation is supported by our finding that improving HRR (for instance, by correcting anemia or reducing inflammation) could be a potential strategy to benefit bone health in adolescents.

Our analysis revealed notable sex-specific differences in the association between HRR and BMD. In general, a higher HRR was significantly associated with greater total BMD in male adolescents, while no significant association was observed in females. Several physiological factors could underlie this disparity. During puberty, males experience a surge in testosterone that increases HGB levels and muscle mass, providing greater mechanical stimulus to bone development. Approximately 65% of the increase in HGB mass in pubertal boys is attributed to rising testosterone levels [46]. Additionally, adolescent boys often engage in more vigorous physical activity than girls, a factor known to enhance BMD [47]. These differences imply that a given HRR value may not have identical implications for bone health in both sexes. A low HRR in a female adolescent might be more likely linked to iron deficiency (with potential direct effects on bone if combined with poor nutrition), whereas in a male, it might signify lower fitness or other health issues affecting bone loading. Adolescent girls are more prone to iron deficiency due to increased iron requirements during growth and menstrual blood loss, which may alter hemoglobin levels and red blood cell indices independent of bone metabolism. Furthermore, variations in menstrual status and cycle regularity may influence both hematological parameters and bone health, thereby attenuating the observed association between HRR and BMD in females. For now, our results highlight the need to account for sex-specific physiology when evaluating blood-based indicators of bone health in adolescence.

Our findings have several clinical implications. The HRR is derived from routine complete blood count parameters and thus can be obtained easily, non-invasively, and at low cost in clinical practice. This positions HRR as a promising potential screening marker for detecting adolescents at risk of suboptimal bone density. Early identification is crucial, as adolescence is a one-time window to build peak bone mass that will determine future osteoporosis risk. Intervention during this critical period can help maximize bone accrual and reduce the likelihood of osteoporosis and fractures later in life. In this context, an adolescent with a low HRR might warrant a closer evaluation of their diet, nutritional status (especially iron, calcium, and vitamin D intake), and lifestyle. For example, a low HRR could prompt screening for iron deficiency or anemia, which, if present, should be treated not only to improve hematologic health but potentially to benefit bone development as well. Similarly, such an individual might be counseled on increasing weight-bearing physical activity and ensuring adequate nutrient intake to support bone growth. Thus, HRR could serve as a simple adjunct risk indicator alongside traditional factors (like BMI, diet, exercise, and menstrual history) in the pediatric or adolescent clinical setting. Moreover, our study adds to the growing evidence that systemic health indicators (inflammation, nutrition, etc.) are intertwined with bone health. For clinicians, this reinforces the importance of a holistic approach: maintaining overall healthy physiology (e.g., preventing chronic inflammation and anemia) may have tangible benefits for the skeleton. While it is premature to recommend broad HRR screening solely for bone health assessment, our results lay the groundwork for considering HRR in comprehensive risk profiles for youth, especially those with other risk factors for low BMD.

This study has several limitations. First, its cross-sectional design precludes causal inference between HRR and BMD, and longitudinal studies are needed to clarify temporal relationships. Second, although we adjusted for multiple demographic, nutritional, and biochemical factors and further assessed covariate balance using standardized mean differences, residual confounding cannot be completely excluded. Third, we did not apply propensity score weighting methods such as inverse probability of treatment weighting (IPTW) or overlap weighting, which might further reduce imbalance. Fourth, information on behavioral and lifestyle characteristics (e.g., physical activity, smoking exposure, breastfeeding history) was not consistently collected across different NHANES cycles, which limited our ability to incorporate these variables. Finally, because NHANES represents U.S. adolescents, the generalizability of our findings to other populations may be limited due to genetic, nutritional, and healthcare differences.

In summary, future investigations should aim to validate HRR as a marker of bone health, elucidate its biological underpinnings, and assess how it can be leveraged in early-life strategies to improve lifelong skeletal well-being.

## 5. Conclusions

This study suggests that the hemoglobin-to-red-cell distribution width ratio is positively associated with bone mineral density in male adolescents. Given that HRR is an inexpensive and routinely available hematological parameter, it may provide supportive information for the preliminary assessment of bone health in clinical or public health settings. However, these findings should be interpreted with caution, and further longitudinal and mechanistic studies are needed before the HRR can be considered as a reliable surrogate marker for bone status.

## Figures and Tables

**Table 1:** Basic characteristics of the study population.

Characteristic	Overall (*n* = 3789)	HRR Quartiles					SMD (Q1 vs. Q4)
		Q1 (*n* = 944)	Q2 (*n* = 948)	Q3 (*n* = 944)	Q4 (*n* = 953)	*p* Value	
Age (years)	15.406 ± 2.237	15.272 ± 2.252	14.932 ± 2.194	15.156 ± 2.210	16.129 ± 2.108	<0.0001	−0.438
Gender (%)						<0.0001	
Male	52.917	26.162	39.83	53.888	81.947		−1.169
Female	47.083	73.838	60.17	46.112	18.053		1.169
Race (%)						<0.0001	
Mexican American	15.561	16.339	14.595	14.332	16.945		−0.115
Other Hispanic	7.68	10.655	8.375	7.411	5.25		0.132
Non-Hispanic White	54.326	37.239	53.807	58.995	62.508		−0.438
Non-Hispanic Black	12.59	24.847	14.944	9.096	5.152		0.580
Other Race	9.844	10.921	8.279	10.165	10.145		−0.123
PIR	2.418 ± 1.574	2.169 ± 1.530	2.434 ± 1.579	2.475 ± 1.557	2.526 ± 1.596	<0.0001	−0.213
BMI (kg/m^2^)	23.965 ± 5.985	24.937 ± 6.796	24.027 ± 6.278	23.193 ± 5.727	23.926 ± 5.199	<0.0001	0.170
HDL-C (mmol/L)	1.339 ± 0.306	1.398 ± 0.325	1.344 ± 0.309	1.357 ± 0.308	1.277 ± 0.275	<0.0001	0.402
BUN (mmol/L)	4.037 ± 1.226	3.806 ± 1.110	4.015 ± 1.309	3.992 ± 1.143	4.258 ± 1.264	<0.0001	−0.364
Total calcium (mmol/L)	9.598 ± 0.297	9.468 ± 0.297	9.574 ± 0.299	9.618 ± 0.264	9.692 ± 0.286	<0.0001	−0.709
Serum 25OHD (nmol/L)	63.065 ± 22.329	58.228 ± 22.816	63.249 ± 22.773	64.898 ± 23.181	64.641 ± 20.239	<0.0001	−0.381
Total BMD (g/cm^2^)	1.030 ± 0.120	1.020 ± 0.114	1.008 ± 0.118	1.016 ± 0.126	1.068 ± 0.113	<0.0001	−0.411
HGB (g/dL)	14.042 ± 1.329	12.426 ± 0.986	13.525 ± 0.613	14.257 ± 0.692	15.421 ± 0.818	<0.0001	−3.324
RDW (%)	13.191 ± 1.076	14.429 ± 1.484	13.221 ± 0.590	12.892 ± 0.597	12.571 ± 0.552	<0.0001	1.660
HRR	1.073 ± 0.141	0.870 ± 0.107	1.023 ± 0.026	1.106 ± 0.025	1.228 ± 0.060	<0.0001	−4.153
Milk product consumption (%)						<0.0001	
Never	6.816	7.483	7.869	6.656	5.589		0.187
Rarely (less than once a week)	10.91	14.601	11.842	9.901	8.429		0.065
Sometimes (once a week or more, but less than once a day	26.697	29.469	26.007	26.536	25.488		−0.222
Often (once a day or more)	55.577	48.448	54.283	56.907	60.493		−0.038

HRR Q1 (<0.9716); HRR Q2 (0.9716–1.0635); HRR Q3 (1.0635–1.1515); HRR Q4 (>1.1515); PIR, Ratio of family income to poverty; BMI (kg/m^2^), Body Mass Index; HDL-C (mmol/L), High-density lipoprotein cholesterol; BUN (mmol/L), Blood urea nitrogen; Total BMD (g/cm^2^), Total bone mineral density; HRR, Hemoglobin-to-red-cell distribution width ratio; HGB (g/dL), Hemoglobin; RDW (%), Red cell distribution width;. Mean ± SD for continuous variables: *p*-value was calculated by a weighted linear regression model. % for categorical variables: *p*-value was calculated by the weighted chi-square test.

**Table 2:** The relationship between HRR and BMD.

	Model 1 (Unadjusted) β (95% CI) *p*-Value	Model 2 (Age, Gender, Race Adjusted) β (95% CI) *p*-Value	Model 3 (Fully Adjusted) β (95% CI) *p*-Value
HRR	0.105 (0.079, 0.131) <0.0001	0.068 (0.043, 0.093) <0.0001	0.078 (0.053, 0.104) <0.0001
Q1	Reference	Reference	Reference
Q2	−0.004 (−0.015, 0.007) 0.445	0.007 (−0.002, 0.016) 0.122	0.008 (−0.001, 0.017) 0.074
Q3	0.004 (−0.007, 0.014) 0.505	0.011 (0.001, 0.020) 0.023	0.014 (0.005, 0.024) 0.003
Q4	0.046 (0.036, 0.057) <0.0001	0.029 (0.019, 0.039) <0.0001	0.033 (0.022, 0.043) <0.0001
*p* for trend	<0.001	<0.001	<0.001

Model 1: No covariates were adjusted. Model 2: Age, gender, and race were adjusted. Model 3: Age, gender, race, ratio of family income to poverty, total calcium, direct HDL cholesterol, serum 25OHD, milk product consumption, BMI, and BUA were adjusted.

**Table 3:** Threshold Effect Analysis of the Association Between the HRR and BMD.

Total BMD	Adjusted β (95% CI) *p* Value
Model I	
Fitting by the standard linear model	0.079 (0.054, 0.105) <0.0001
Model II	
Fitting by the standard linear model	
Inflection point (K)	1.055
HRR < K	0.039 (0.002, 0.076) 0.041
HRR > K	0.143 (0.094, 0.193) <0.0001
Log likelihood ratio	0.003

Age, gender, race, ratio of family income to poverty, total calcium, HDL-C, serum 25OHD, milk product consumption, BMI and BUN were adjusted.

## Data Availability

The survey data are publicly available on the Internet for data users and researchers throughout the world (www.cdc.gov/nchs/nhanes/, accessed on 10 March 2025).

## Abbreviations

PIR, Ratio of family income to poverty; BMI (kg/m^2^), Body mass index; HDL-C (mmol/L), High-density lipoprotein cholesterol; BUN (mmol/L), Blood urea nitrogen; Total BMD (g/cm^2^), Total bone mineral density; HRR, Hemoglobin-to-red-cell distribution width ratio; HGB (g/dL), Hemoglobin; RDW (%), Red cell distribution width; NHANES, National Health and Nutrition Examination Survey; GAM, Generalized additive model.
